# Melatonin Activates Phenylpropanoid Metabolism and Antioxidant Defense to Preserve Quality of Fresh-Cut Potatoes During Cold Storage

**DOI:** 10.3390/foods15071234

**Published:** 2026-04-04

**Authors:** Xingyue Ma, Hao Wang, Xiju Wang, Xingyu Li, Hui Li, Dongqing Wang, Yang Yang

**Affiliations:** 1 Laboratory of Plant Resources Preservation and Processing, College of Food Science and Engineering, Inner Mongolia Agricultural University, Hohhot 010018, China; maxingyue132@163.com (X.M.); 19847362253@163.com (H.W.); 18747644675@139.com (X.W.); 15547102067@163.com (X.L.); 2 Inner Mongolia HUARUI Inspection and Testing Co., Ltd., Intelligent Manufacturing Industrial Park D3-1, Hulindere New District, Hohhot 011500, China; 13948317213@163.com; 3 Inner Mongolia Fresh Potato Breeding and Farming Farmers’ Professional Cooperative, No. 152, Maoshengying Agricultural Park, Jinhe Town, Saihan District, Hohhot 010030, China; m18196399222@163.com

**Keywords:** melatonin, fresh-cut potatoes, phenylpropanoid metabolism, ethylene, refrigerated quality

## Abstract

To develop safe and effective preservatives for fresh-cut produce, this study elucidates the multi-pathway mechanisms through which Melatonin (MT) regulates postharvest senescence in fresh-cut potatoes. Treatment with 0.1 mmol/L exogenous MT effectively inhibited browning and softening during storage. In terms of browning control, MT suppressed PPO and POD activities by 46% and ~10% at the end of storage (day 12), while enhancing enzymatic and non-enzymatic antioxidant capacity by 1.1- to 1.6-fold on average throughout storage. This alleviated oxidative damage and membrane lipid peroxidation, thereby reducing tissue browning. Regarding texture maintenance, MT downregulated PME and cellulase activities by 23% and 19% at the end of storage, activated phenylpropanoid metabolism, and inhibited starch degradation (maintaining 19% higher starch content), thus preserving cell wall structure and firmness (9.2% higher at the end of storage). Further analysis revealed that MT antagonized ethylene biosynthesis, upregulated *StMYB168* expression (5.8-fold higher than control on average), and activated endogenous MT biosynthesis, establishing a self-sustaining positive regulatory cycle. Correlation analysis confirmed close relationships among physiological processes, signaling responses, and quality traits, with significant associations between firmness and starch content (r = 0.72), color indices and PPO/POD (|r| > 0.65), and MT biosynthesis genes and metabolic pathways (r = 0.65–0.75) (*p* < 0.01).

## 1. Introduction

The global consumption of fresh-cut potatoes is steadily increasing, yet their shelf life remains constrained by quality issues such as browning, softening, and microbial proliferation. While chlorine-based disinfectants can partially suppress microbial growth and delay enzymatic browning, their efficacy in preventing softening is limited. Additionally, these disinfectants may generate toxic by-products, including chloroform and trichloromethane, leading to their prohibition in certain European countries [1].

Melatonin (MT) is an indoleamine compound naturally present in animals and plants. In recent years, it has been demonstrated to play a role in regulating not only the physiological rhythms of animals but also plant physiological processes such as stress defense, delayed senescence, and hormonal balance regulation [2,3,4]. These properties render it highly promising for application in the field of fruit and vegetable preservation, where its efficacy has been confirmed in various fruits and vegetables, including lychees [5] and strawberries [6]. Fresh-cut produce represents metabolically active plant tissues that maintain cellular-level metabolic regulation. This characteristic provides a theoretical foundation for intervening in physiological processes and delaying quality deterioration through exogenous signal molecules like MT, with a preservation mechanism distinct from traditional disinfectants and bacteriostatic agents. Prior studies have indicated that MT can enhance the quality of fresh-cut potatoes [7].

The quality decline of fresh-cut potatoes arises from the synergistic effects of mechanical wounding and postharvest senescence. Enzymatic oxidation of polyphenols induces browning [8], while the reduction in cell wall mechanical strength and starch degradation lead to softening [9]. Additionally, oxidative stress-induced depletion of antioxidants such as ascorbic acid contributes to nutrient loss. Physiologically, these changes are linked to enhanced ethylene release and accelerated senescence triggered by cutting [10,11]. The antioxidant benefits of MT for quality enhancement have been demonstrated in fresh-cut broccoli [12], star fruit [13], and sweet potatoes [14], and preliminary studies on potatoes have been published.

However, existing research on MT-treated fresh-cut potatoes has primarily focused on browning control while largely overlooking traumatic stress responses specific to cutting injury. Furthermore, the regulatory functions of MT in ethylene production, senescence delay, and cell wall reinforcement in this specific crop remain inadequately explored. Consequently, the comprehensive physiological mechanisms underlying MT-mediated quality maintenance in fresh-cut potatoes are not fully understood.

## 2. Materials and Methods

### 2.1. Materials

Potatoes (*Solanum tuberosum* ‘Jizhang 12’) were cultivated at a commercial farm in Jining District, Ulanqab, Inner Mongolia, and harvested at 96 days after emergence when >75% of the foliage had senesced. Uniform, healthy tubers (150–200 g) free from visible defects, mechanical injury, or deformation were selected for the experiment.

### 2.2. Test Methods

#### 2.2.1. Fresh-Cut Processing Methodology

Following IFPA guidelines [15], tubers were washed, peeled, sliced to 3 mm thickness, and treated accordingly. Slices were soaked for 10 min in 0.05, 0.1, or 0.5 mmol/L MT or distilled water (control), drained, vacuum-packed in polyethylene (PE) bags (0.14 mm thickness, oxygen permeability: 3.5 × 10^−3^ mL·m^−2^·day^−1^·atm^−1^ at 23 °C, 0% RH; water vapor permeability: 1.2 g·m^−2^·day^−1^ at 38 °C, 90% RH), and stored at 4 ± 0.5 °C and 85–90% relative humidity in darkness. Analyses were performed on days 0, 3, 6, 9, and 12, with three independent biological replicates per treatment (*n* = 3).

#### 2.2.2. Appearance Quality Attributes

L* values and browning index (BI). L*, a*, and b* values were measured at three random points per slice. BI was calculated via the following formula [16]:

$$BI=\frac{\left(x-0.31\right)\times 100}{0.172}$$

$$x=\frac{a*+1.75\times L*}{5.645\times L*+a*-3.012\times b*}$$

Firmness. Texture profile analysis (TPA) parameters were: P36R probe (36 mm diameter), pre-test speed 2.0 mm/s, test speed 1.0 mm/s, post-test speed 2.0 mm/s, trigger force 5 g, and compression strain 20%. For each slice, hardness was measured at three anatomical regions (cortex, vascular ring, and pith; *n* = 3 per region). The mean of nine measurements per treatment (3 slices × 3 regions) was calculated as the final hardness value [17].

#### 2.2.3. Scanning Electron Microscopy (SEM) Sample Preparation and Observation

Sample cubes (5 × 5 × 3 mm) were fixed in 2.5% glutaraldehyde, buffer-washed, ethanol-dehydrated, treated with hexamethyldisilazane (HMDS), gold-coated, and imaged via a Hitachi Regulus 8220 SEM (Hitachi, Tokyo, Japan) at 15 kV [18].

#### 2.2.4. Enzymatic Assays Related to Quality Attributes

Extraction procedure and unit. Unless otherwise specified, 1 g of tissue was homogenized in 5 mL ice-cold extraction buffer and centrifuged (10,000× *g*, 10 min, 4 °C). The supernatant was used as the enzyme extract. Enzyme activities are expressed as U·g^−1^·FW, where one unit (U) is defined for each enzyme in Table 1.

Assay conditions. Polyphenol oxidase (PPO), peroxidase (POD), pectin methylesterase (PME), cellulase (Cx), phenylalanine ammonia-lyase (PAL), 4-coumarate:CoA ligase (4CL), cinnamate-4-hydroxylase (C4H), superoxide dismutase (SOD), catalase (CAT), ascorbate peroxidase (APX), and glutathione reductase (GR) activities were determined following established methods as detailed in Table 1.

#### 2.2.5. Metabolite Quantification

Lignin content. Lignin content was analyzed via thioacidolysis-HPLC-DAD [22] with three key modifications for starch-rich potato tissue—(1) starch elimination: cell wall residues (from 2 g tissue) underwent α-amylase digestion (1000 U/mL, 85 °C, 2 h, pH 6.0) following sequential solvent washes, with complete removal verified by IKI staining; (2) optimized thioacidolysis: starch-free residues (20–30 mg) reacted with BF_3_·Et_2_O (dioxane:ethanethiol, 8.75:1, *v*/*v*, 100 °C, 4 h) under nitrogen; and (3) enhanced purification: CH_2_Cl_2_ extraction (3 × 2 mL), nitrogen drying, and C18 HPLC separation (methanol/0.1% phosphoric acid gradient, 280 nm detection). Total lignin (µg·g^−1^ FW) was summed from coniferyl, p-coumaryl, and sinapyl alcohols via external standards.

Carbohydrates and MDA. Reducing sugars (DNS method) and malondialdehyde (MDA, thiobarbituric acid method) were assayed spectrophotometrically [19].

Other metabolites. Starch, AsA, and total phenolics (TP) were extracted and quantified using commercial kits (Nanjing Jiancheng Bioengineering Institute, Nanjing, China) following acid hydrolysis [19], 1,10-phenanthroline reaction [19], and the ammonium molybdate method [23], respectively.

#### 2.2.6. Oxidative Stress Parameters

The radical scavenging capacity was assessed via DPPH and ABTS assays with slight modifications [24]. Briefly, the samples were extracted with 80% methanol and centrifuged. For DPPH, the extract was reacted with 0.1 mM DPPH ethanol solution in the dark, and the absorbance was measured at 517 nm. For ABTS, the radical cation solution was prepared by oxidizing ABTS with potassium persulfate and then mixing with the extract and measuring at 734 nm. The scavenging activity (%) was calculated as (Acontrol − Asample)/Acontrol × 100 for both methods.

Membrane integrity. The ion permeability was determined via the electrolyte leakage method [19].

#### 2.2.7. Hormone and Gene Analysis

Ethylene biosynthesis. The endogenous ethylene release was determined via GC (GC-2014C, Shimadzu, Shanghai, China) via the method described on page 108 of reference [19]. The samples were analyzed after removal of their packaging via an FID detector and a stainless-steel packed column with the oven temperature set at 70 °C.

ACS and ACO activities were determined via ELISA via commercial kits (Beijing Ruishuo Biotechnology Co., Ltd., Beijing, China) according to the manufacturer’s instructions [25].

MT biosynthesis. MT content and serotonin N-acetyltransferase (SNAT) and tryptamine 5-hydroxylase (T5H) activities were quantified via ELISA kits (Suzhou Grace Biotechnology, Suzhou, China) [26].

Gene expression analysis. Total RNA was extracted (Tiangen RNA prep Kit, Cat. #DP441; Tiangen Biotech, Beijing, China) and reverse-transcribed (Vazyme HiScript III RT SuperMix; TransGen Biotech Co., Ltd., Beijing, China) following manufacturer protocols. qPCR (TransStart Green qPCR SuperMix; TransGen Biotech Co., Ltd., Beijing, China) was performed on a CFX96 system (Bio-Rad; Bio-Rad Laboratories, Hercules, CA, USA) with primers (5′→3′): Stef1a (Accession: AB061263, F: ATTGGAAACGGATATGCTCCA; R: TCCTTACCTGACGCCTGTCA) and *StMYB168* (Accession: XM_006357647.2, F: CAGACGATTTATCCACGAG; R: TTGGCATTCCACTACTTCA). Biological triplicates were analyzed using the 2^−ΔΔCT^ method [27].

### 2.3. Statistical Analysis

For enzyme activity assays, metabolite quantification, and gene expression analysis, three biological replicates were conducted for each measurement. Data were expressed as means ± standard error (SE) from three independent biological replicates (*n* = 3). One-way ANOVA followed by Duncan’s multiple range test (post hoc) was applied to compare means among treatments at each time point using SPSS 26.0 (IBM Corp., Armonk, NY, USA). Statistical significance was defined at *p* < 0.05. Homogeneity of variance was tested using Levene’s test prior to ANOVA. Data visualization was performed in Origin 2024. Pearson correlation analyses were conducted on the Lianchuang Bioinformatics Cloud Platform, with correlation matrices displayed as heatmaps; final figure assembly and editing were completed in Inkscape 1.0.

## 3. Results and Analyses

### 3.1. Effect of MT Treatment on L* Value and BI Values of Fresh Cut Potatoes

**Figure 1:**
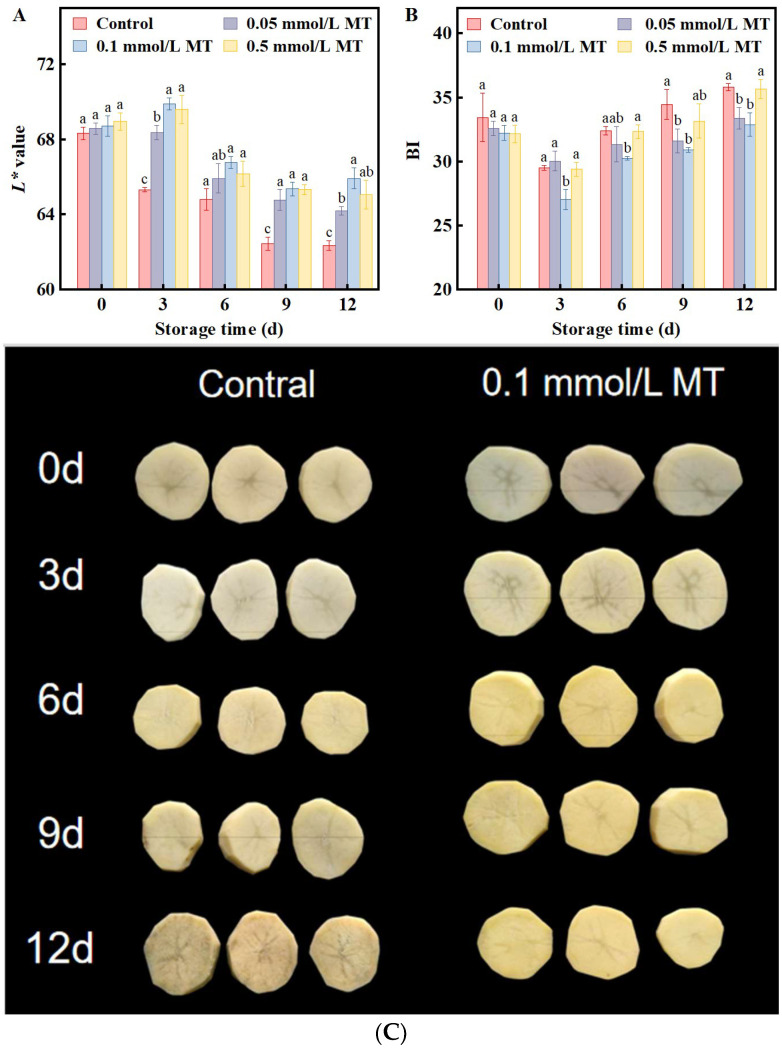
Effect of different concentrations of MT (0, 0.05, 0.1, and 0.5 mmol/L) on L* value (**A**), BI (**B**) and appearance (**C**) of fresh-cut potatoes during cold storage at 4 °C. Values are means ± SD (*n* = 3). Different lowercase letters above bars within the same timepoint indicate significant differences among concentrations (*p* < 0.05).

Color retention is the most direct indicator of quality in fresh-cut potatoes [8]. The L* value, an objective measure of browning (lower values indicate darker color and greater browning), decreased continuously in the control group over 12 d of storage. MT at all the tested concentrations significantly attenuated this decrease, with L* values remaining higher than those of the control throughout the observation period (*p* < 0.05). Among the treatments, 0.1 mmol/L MT had the highest L* value at every sampling time, significantly outperforming 0.05 mmol/L at day 3 and day 12 (*p* < 0.05), while showing a numerically higher but statistically similar value compared to 0.5 mmol/L (*p* > 0.05) (Figure 1A).

The browning index (BI) is an important parameter for quantifying browning severity in fresh-cut potatoes: the higher the BI value, the more pronounced the browning. While the browning index (BI) rose in all groups, the degree of increase was not uniform across all treatment groups and the control (Figure 1B). Compared with the control, 0.05 mmol/L MT significantly reduced the BI on days 9 and 12, whereas 0.5 mmol/L MT produced only a modest, nonsignificant reduction on day 9. However, under the 0.1 mmol/L MT treatment, the BI was significantly lower than that of the control throughout storage; by day 12, the BI was 91.87% of the control value (*p* < 0.05). Notably, 0.1 mmol/L MT exhibited a significantly lower BI than 0.05 mmol/L on day 3 (*p* < 0.05), with numerically lower but statistically similar values at other sampling points (*p* > 0.05). In contrast, 0.1 mmol/L MT consistently outperformed 0.5 mmol/L, showing significantly lower BI values at all time points except day 9, where the difference was numerical but not statistically significant (*p* > 0.05).

### 3.2. Effect of MT Treatment on Microstructure and Hardness of Fresh-Cut Potatoes

**Figure 2:**
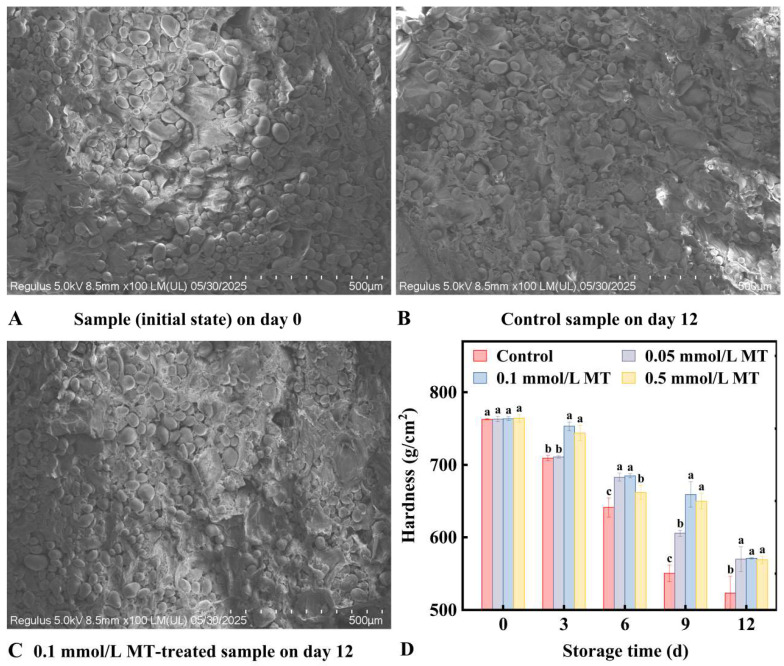
Effects of MT on the microstructure (**A**–**C**) and hardness (**D**) of fresh-cut potatoes. (**A**) Day 0 electron microscopy; (**B**) day 12 control electron microscopy; (**C**) day 12 0.1 mmol/L treatment electron microscopy; and (**D**) hardness of samples treated with 0, 0.05, 0.1, and 0.5 mmol/L MT. Values are means ± SD (*n* = 3). The bars represent the standard deviation of the mean, and different letters above the bars within the same time indicate a significant difference at the level of *p* < 0.05 level.

Hardness serves as an indicator of freshness and is closely tied to the overall quality of fresh-cut potatoes. As shown in Figure 2D, the hardness of fresh-cut potatoes generally decreased during storage, but all MT concentrations inhibited this decrease to varying degrees. Specifically, the 0.1 mmol/L MT treatment group exhibited significantly higher hardness than the 0.05 mmol/L group on days 3 and 9 (*p* < 0.05), with numerically higher but statistically similar values at other sampling points (*p* > 0.05). Similarly, compared with the 0.5 mmol/L group, 0.1 mmol/L MT showed a significantly higher hardness only on day 6 (*p* < 0.05), while remaining numerically superior but statistically indistinguishable at other time points (*p* > 0.05). Overall, the 0.1 mmol/L MT treatment group maintained the highest average hardness among all groups throughout the storage period. At day 12 (end of storage), the hardness of 0.1 mmol/L MT treatment group was 9.1% higher than that of the control. The hardness of fresh potato tissue is intricately linked to its integrity. Hardness reflects tissue integrity (Figure 2). We compared cell structures at day 0 (A) and after 12 days of storage for control (B) and 0.1 mmol/L MT-treated potatoes (C). Freshly cut potatoes exhibit clear cell wall structures and intact starch granules. After 12 days of storage, the cell structure sustains partial damage, starch granules dissolve and diffuse, and cell wall tissues fragment. A comparison of the microscopic structural changes in potatoes during storage revealed more severe tissue deformation and starch granule dissolution in the control group. By day 12, the control group’s cell organization was entirely disordered, whereas changes in the 0.1 mmol/L MT treatment group were relatively minor.

### 3.3. Effect of 0.1 mmol/L MT Treatment on PPO, POD, PME and Cellulase Activities of Fresh Cut Potatoes

**Figure 3:**
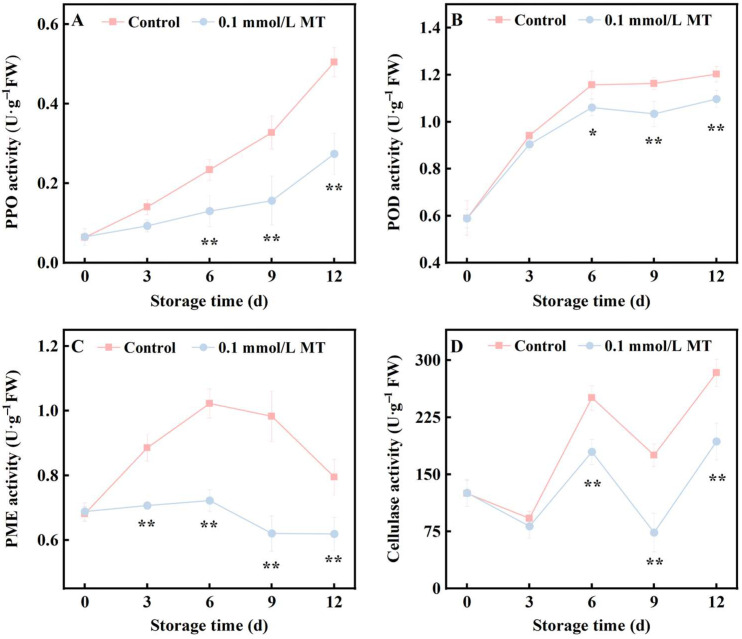
Effects of different concentrations of MT on the PPO (**A**), POD (**B**), PME (**C**) and Cx (**D**) activities of fresh-cut potatoes during cold storage at 4 °C. Values are means ± SD (*n* = 3). The vertical bars represent the standard deviations of the values of triplicate assays. * Denotes between-group differences at the same time point with *p* < 0.05; ** denotes between-group differences at the same time point with *p* < 0.01.

PPO and POD are the enzymes most associated with browning. Figure 3A,B shows that as the storage time increased, the activities of both PPO and POD in fresh-cut potatoes increased. However, in the 0.1 mmol/L MT treatment group (hereafter referred to as the treatment group), the increase in PPO and POD activities was slower than that in the control group. Notably, beginning on the 6th day of storage, the PPO and POD activities in the treatment group were significantly lower than those in the control group (*p* < 0.01). At day 12, PPO and POD activities were reduced by 46% and ~10%, respectively, compared to the control.

PME and Cx are enzymes involved in cell wall metabolism. PME breaks down pectin to weaken the middle lamella, whereas Cx degrades cellulose to reduce the strength of the primary wall. Both processes affect plant tissue stability. Figure 3C shows that in the control group, the PME activity initially increased but then decreased during storage, peaking on day 6. In contrast, the treatment group presented consistently lower PME activity without a distinct peak throughout the storage period. Statistical analysis indicated that the PME activity in the treatment group was significantly lower than that in the control group (*p* < 0.01) throughout the entire storage period, averaging 23% lower than the control across all sampling points. Figure 3D indicates that both groups presented fluctuating increasing trends in Cx activity during storage. However, starting from day 6 of storage, the Cx activity in the treatment group was significantly lower than that in the control group (*p* < 0.01) and was, on average, 39% lower throughout the subsequent storage period.

### 3.4. Effect of MT Treatment on PAL, C4H, 4CL and Lignin in Fresh-Cut Potatoes

**Figure 4:**
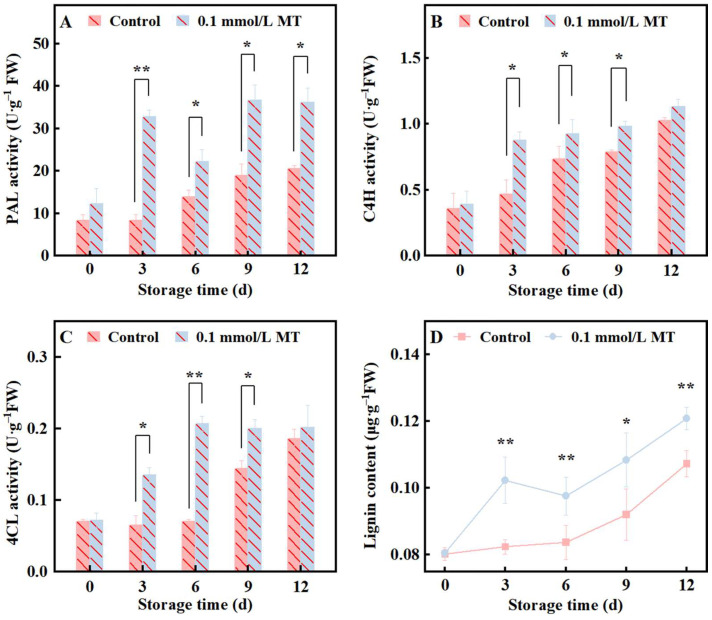
Effects of different concentrations of MT on the PAL (**A**), C4H (**B**), and 4CL (**C**) activities and lignin contents (**D**) of fresh-cut potatoes during cold storage at 4 °C. Values are means ± SD (*n* = 3). The vertical bars represent the standard deviations of the values of triplicate assays. * Denotes between-group differences at the same time point with *p* < 0.05; ** denotes between-group differences at the same time point with *p* < 0.01.

The phenylpropanoid metabolic pathway is crucial for plant stress resistance and overcoming mechanical damage [28]. Figure 4A–C shows that PAL, C4H and 4CL activities in fresh-cut potatoes all increased to varying degrees during storage, but the increase was greater in the treatment group. PAL activity in the treatment group was significantly higher than that in the control group throughout the storage period, and C4H and 4CL activities were significantly higher than those in the control group from the 3rd to the 9th day of storage. When averaged across all sampling points, PAL, C4H, and 4CL activities in the MT group were 2.0-, 1.3-, and 1.6-fold higher than the control, respectively (*p* < 0.01). Lignin is one of the main end products of the phenylpropanoid metabolic pathway and is closely related to the formation of secondary cell walls and tissue hardness in plants. Figure 4D shows that the lignin content of fresh-cut potatoes increased during storage. The increase was more rapid in the treatment group, which was significantly higher than the control throughout the storage period. When averaged across all sampling points, lignin content in the MT group was 1.1-fold higher than in the control (*p* < 0.05). The above results suggest that cutting may lead to the passive upregulation of the phenylpropanoid metabolic pathway, but MT treatment may increase the lignin content in cells by actively upregulating the phenylpropanoid metabolic pathway, thereby affecting product hardness.

### 3.5. Effects of 0.1 mmol/L MT on Carbohydrate Metabolism, Antioxidant System, and Membrane Integrity of Fresh-Cut Potatoes

**Figure 5:**
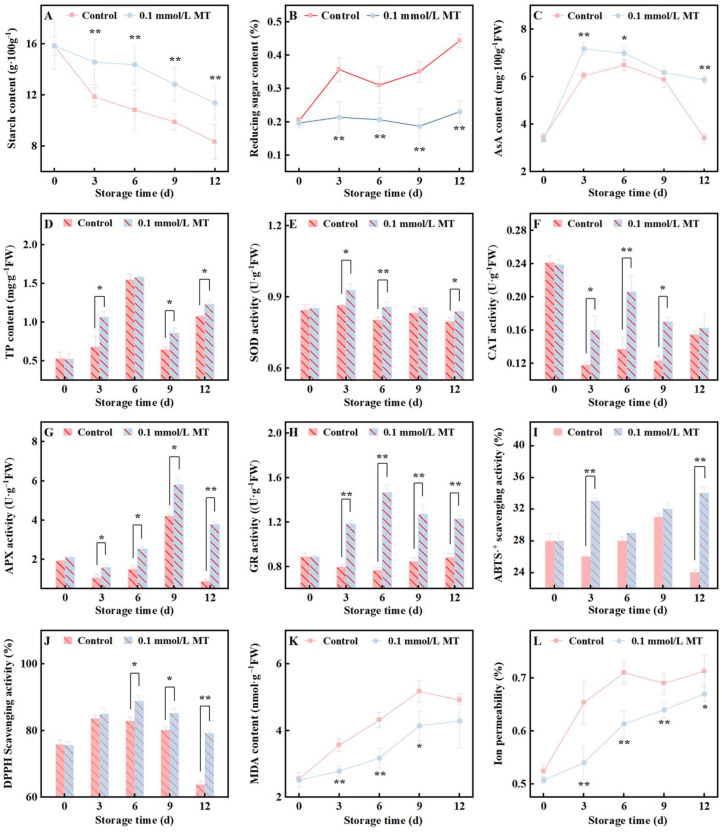
Effects of different concentrations of MT on starch content (**A**), reducing sugar content (**B**), AsA content (**C**), total phenolics (TP) content (**D**), superoxide dismutase (SOD) activity (**E**), catalase (CAT) activity (**F**), APX activity (**G**), glutathione (GR) activity (**H**), ABTS+ scavenging activity (**I**), DPPH+ scavenging activity (**J**), malondialdehyde (MDA) content (**K**), and ion permeability (**L**) of fresh-cut potatoes during cold storage at 4 °C. Values are means ± SD (*n* = 3). The vertical bars represent the standard deviations of the values of triplicate assays. * Denotes between-group differences at the same time point with *p* < 0.05; ** denotes between-group differences at the same time point with *p* < 0.01.

Starch is both the chief nutritional indicator of a fresh-cut potato and the primary determinant of its firmness. Mechanical cutting triggers elevated ethylene production and respiration, accelerating starch consumption and leading to its rapid decline. Figure 5A shows that starch content decreased to 53% of the initial value in the control group after 12 days, whereas the treatment group retained 72% and was significantly higher throughout storage (*p* < 0.05). Reducing sugars, the principal products of starch hydrolysis, accumulated to 2.2-fold initial levels in the control group by day 12 (Figure 5B). In contrast, the treatment group showed only minor fluctuations and remained significantly lower than the control throughout storage (*p* < 0.05).

AsA and total phenolics are the major nonenzymatic antioxidants that respond to abiotic stresses. As shown in Figure 5C,D, both AsA and total phenolics in the control group increased transiently during the first 6 d and then declined. This transient rise is attributable to the wounding response, whereas the subsequent decrease reflects gradual loss of cellular integrity, exacerbated oxidative injury and diminished biosynthetic capacity. Although wounding responses are generally detectable within 12 h, the modified atmosphere and vacuum packaging employed here likely delayed the onset of wounding. The treatment group maintained AsA and total phenolic levels higher than the control group throughout storage (AsA was significantly higher on days 3, 6, and 12, and phenolics on days 3, 9, and 12; *p* < 0.05). When averaged across all sampling points, AsA and phenolic contents in the treatment group were 1.2- and 1.1-fold higher than those in the control group, respectively (*p* < 0.05).

Antioxidant enzymes are the primary defense against oxidative stress. The activities of SOD, CAT, APX, and GR were therefore monitored as representative indicators. Figure 5E–H shows that MT treatment increased all four enzyme activities, albeit to varying degrees. The treatment group exhibited significantly higher SOD activity than the control group on days 3, 6, and 12, and higher CAT activity on days 3, 6, and 9 (see Figure 5E,F for detailed statistical comparisons). APX and GR activities in the treatment group remained consistently higher than those in the control group from day 3 onward (see Figure 5G,H for detailed statistical comparisons). When averaged across all sampling points, CAT, APX, and GR activities in the treatment group were significantly higher than those in the control group (1.3-, 1.6-, and 1.4-fold higher, respectively; *p* < 0.05) (Figure 5E–H).

ABTS·^+^ and DPPH· scavenging activities, used to assess antioxidant capacity, were both higher in the treatment group than the control group throughout storage, with significant differences on days 3 and 12 for ABTS·^+^ and on days 6 and 12 for DPPH· (see Figure 5I,J for detailed statistical comparisons). When averaged across all sampling points, ABTS·^+^ was 1.1-fold higher in the treatment group (*p* < 0.05).

MDA accumulates as senescence progresses. It is an irreversible end-product of membrane lipid peroxidation. The MDA content increased continuously in both treatment groups, but the rate was markedly lower in the treatment group than in the control group throughout storage (*p* < 0.05, Figure 5K).

Electrolyte leakage reflects the loss of membrane semipermeability and hence cellular senescence. The electrolyte leakage increased progressively in the control group and peaked on day 9 at 1.4-fold the initial value (Figure 5L). MT treatment significantly attenuated this increase: on day 12, the electrolyte leakage in the treatment group increased to 1.2-fold the initial value and remained significantly lower than that in the control group throughout the storage period (*p* < 0.05).

### 3.6. Effects of 0.1 mmol/L MT Treatment on Ethylene Biosynthesis, MT Biosynthesis, and StMYB168 Relative Expression in Fresh-Cut Potatoes

**Figure 6:**
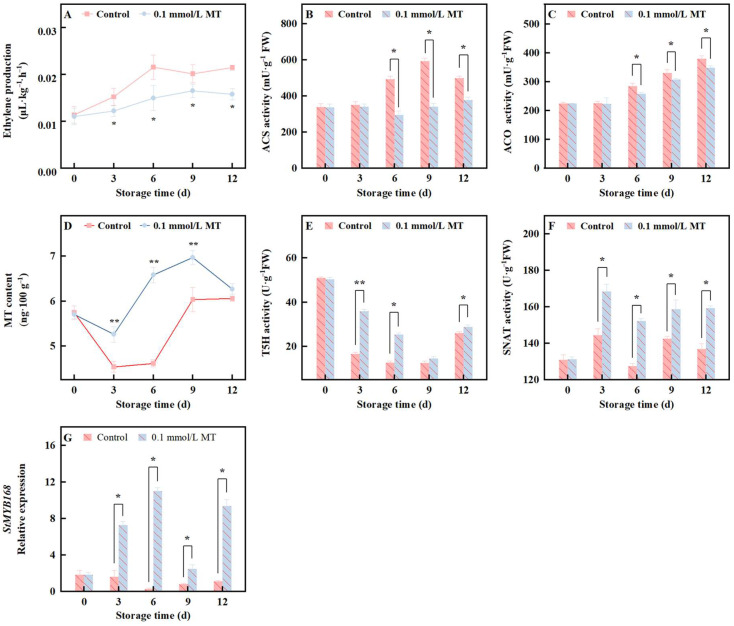
Effects of different concentrations of MT on ethylene release (**A**), ACS (**B**), ACO (**C**), MT content (**D**), T5H (**E**), SNAT activity (**F**) and *StMYB168* (**G**) in fresh-cut potato during cold storage at 4 °C. Values are means ± SD (*n* = 3). The vertical bars represent the standard deviations of the values of triplicate assays. * Denotes between-group differences at the same time point with *p* < 0.05; ** denotes between-group differences at the same time point with *p* < 0.01.

Stress factors induce ethylene, which accelerates senescence in plant tissues and organs. As shown in Figure 6A, the endogenous ethylene release of fresh-cut potatoes increased significantly with prolonged storage time, peaking on the 6th day of storage. Similarly, the activities of ACS and ACO, two key enzymes in the ethylene biosynthesis pathway, also tended to increase during the storage period (Figure 6B,C). The treatment group exhibited significantly lower endogenous ethylene release and ACS and ACO activities throughout the storage period (see Figure 6A–C for detailed statistical comparisons). These results collectively suggest that cutting injury exacerbates the senescence of fresh-cut potato tissues by promoting endogenous ethylene synthesis.

Endogenous MT synthesis in plants is modulated by multiple cues, with adverse conditions being the most common trigger. In the control group, however, the MT content of fresh-cut potatoes decreased sharply from day 3 to day 6 and then rebounded on day 9 to the level observed immediately after cutting (no significant difference from day 0; Figure 6D). Concomitantly, T5H activity—central to the MT pathway—declined progressively until day 12 (Figure 6F). This decline likely reflects physical disruption of the endoplasmic reticulum by cutting, causing loss of cellular contents and transient deactivation of membrane-bound T5H; activity later recovers as wounded tissues undergo self-repair. In the treatment group, MT levels decreased on day 3 but remained significantly greater than those in the control group thereafter, indicating an early contribution of exogenous MT. Throughout storage, the T5H activity in the treatment group remained significantly elevated compared with that in the control group on days 3, 6 and 12, particularly on days 3 and 6, reaching 2.2- and 2.0-fold higher, respectively (*p* < 0.05). SNAT activity in the control group fluctuated without a clear trend, whereas the MT-treated group presented consistently higher SNAT activity at every sampling point (*p* < 0.05, Figure 6F).

Figure 6G shows that, in the control group, the transcript abundance of *StMYB168*, a key transcription factor for lignin biosynthesis, decreased sharply from day 6 of storage onward. In contrast, the treatment group presented strong and sustained upregulation of *StMYB168*, reaching levels that were, on average, 5.8-fold greater than those of the control group throughout the storage period.

**Figure 7:**
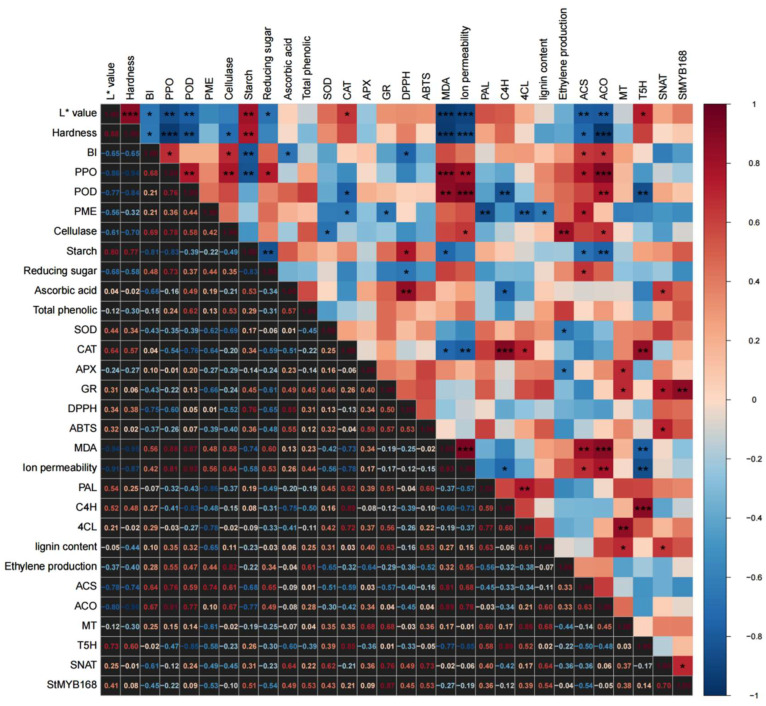
Correlation analysis. * *p* < 0.05, ** *p* < 0.01, *** *p* < 0.001.

Figure 7 presents a comprehensive correlation matrix of all the measured parameters. Associations (|r| > 0.65) [29] were detected between color-related indices (L*, browning index, BI) and the activities of PPO and POD; L* exhibited significant negative correlations with BI, PPO and POD (*p* < 0.01). Tissue firmness was positively correlated with starch content (r = 0.72, *p* < 0.001) and negatively correlated with Cx activity (r = −0.68, *p* < 0.001). Color and textural traits were further associated with membrane deterioration markers—MDA accumulation, ion leakage and ethylene biosynthesis—with correlation coefficients ranging from 0.60 to 0.78 (*p* < 0.01). Notably, genes involved in MT biosynthesis presented robust positive correlations with the antioxidant capacity, phenylpropanoid flux and transcript abundance of *StMYB168* (r = 0.65–0.75, *p* < 0.01), underscoring the coordinated regulation of color, texture and stress responses.

## 4. Discussion

As a versatile signaling molecule, MT has garnered significant attention in recent years because of its pivotal role in enhancing the storage quality of postharvest fruits and vegetables [2]. Previous studies have demonstrated its ability to mitigate browning in fresh-cut potatoes by suppressing the activities of the polyphenols PPO and POD [7]. Building on these findings, this study comprehensively analyzed the mechanisms by which exogenous MT enhances the quality of fresh-cut potatoes from multiple perspectives. Our results revealed that the ability of MT to improve quality extends beyond merely reducing the activity of browning enzymes. 

Triggered by the defense mechanisms of plants in response to stress factors including mechanical wounds [30], browning serves as a prominent indicator of quality deterioration in fresh-cut potatoes. Specifically, it occurs due to the polymerization of quinone compounds, which are formed through the oxidation of polyphenolic substances by enzymes in the presence of oxygen. The physiological role of browning involves leveraging the antibacterial properties and cross-linking effects of quinone compounds to inhibit the proliferation of pathogenic microorganisms on the wound surface and establish a physical barrier [31]. However, the browning of fresh-cut fruits and vegetables significantly diminishes their commercial value by negatively impacting both the color and flavor of the products [32]. While this absolute reduction may appear modest, it corresponds to a visually perceptible difference in browning severity that significantly impacts consumer acceptance. Previous studies indicate that BI differences >5% are detectable by consumers and influence purchasing decisions. Furthermore, the cumulative effect of this reduction over the 12-day storage period translates to extended commercial shelf-life by approximately 2–3 days, providing substantial economic value for the fresh-cut potato industry [33]. The activity of enzymes is a direct factor affecting the browning process. PPO is the most prevalent enzyme responsible for browning in plants. Existing studies tend to demonstrate that MT can inhibit PPO activity in fruits and vegetables [7], a finding that is corroborated by our results. Existing studies generally indicate that the inhibition of PPO by MT likely occurs at the transcriptional level rather than through direct interaction with proteins [34]. The types of reactions catalyzed by POD in plants are more diverse than those catalyzed by PPO [35]. Therefore, the response patterns of POD to MT treatment vary across different studies. While most studies related to the disease resistance of fruits and vegetables have demonstrated that the positive effects of MT are associated with increased POD activity [36,37,38], investigations concerning fresh-cut fruits and vegetables predominantly revealed an inhibitory effect of MT on POD activity [14,34,39], a conclusion that was also supported by our research. This discrepancy could be attributed to the distinct contact processes between the MT treatment solution and intact fruits versus cut tissues. In cut tissues, direct exposure to MT theoretically allows MT to interact directly with the active center of POD or certain hydrophilic groups, potentially leading to POD inactivation.

Firmness is a critical quality indicator for fresh-cut potatoes and is influenced by a combination of factors, including raw material characteristics, the water loss rate, and microbial reproduction. Under identical processing and preservation conditions, the cell wall strength and starch content of raw materials are the most significant factors affecting the hardness of fresh-cut potatoes, providing structural support to the tissue of potato slices [9,38,40]. The plant cell wall, from the outermost layer to the innermost layer, consists of the middle lamella, primary wall, and secondary wall, with the corresponding characteristic constituents being pectin, cellulose, and lignin, respectively. Numerous studies have reported that MT enhances the hardness of plant tissues and organs by reinforcing cell wall structures. For example, in postharvest fruits such as strawberry, pear [41], and plum [42], MT inhibits the activities of pectinase and Cx enzymes, such as PME, thereby delaying the degradation of pectin and cellulose [43]. Additionally, MT strengthens the mechanical support and stress resistance of plants by promoting lignin synthesis. Key enzymes in the phenylalanine metabolic pathway, such as PAL, C4H, and 4CL, are crucial for responding to MT activity regulation [37,44,45]. For starchy fruits and vegetables, such as potato, small lily bulbs and Hami melons, the starch content is another crucial factor influencing hardness [46,47]. MT maintains product hardness by inhibiting starch degradation. In this study, scanning electron microscopy revealed that in the MT-treated group, the starch granule structure was relatively clear, and the cell wall structure was relatively intact (Figure 2A). This finding is closely associated with the reduced activities of PME and Cx (Figure 3C,D). MT significantly upregulated the activities of PAL, C4H, and 4CL. This in turn promoted lignin accumulation (Figure 4). Notably, as shown in Figure 5A,B,D, MT treatment inhibited starch degradation in fresh-cut potatoes, reduced the levels of its degradation products (reducing sugars), and increased the content of phenolic compounds, which are monomeric precursors of lignin. On the basis of these findings, we propose that MT enhances the firmness of fresh-cut potatoes through two mechanisms: (1) inhibiting starch degradation and (2) reinforcing the mechanical strength of the cell wall. The correlation analysis (Figure 7) further supports our hypothesis. When averaged across all sampling points, lignin content in the MT group was 1.1-fold higher than in the control (*p* < 0.05). Although this relative increase appears modest, lignin deposition in fresh-cut potatoes occurs primarily in the wound-healing tissue (approximately 10–15% of the total tissue volume). Localized accumulation at the wound surface creates a reinforced physical barrier that significantly enhances tissue mechanical strength. Our SEM observations (Figure 2C) support this, showing preserved cell wall integrity in MT-treated samples. The 1.1-fold increase in total lignin content translates to an estimated 7–10% increase in wound-specific lignin concentration, sufficient to substantially improve tissue firmness.

The relationship between senescence and the deterioration of quality in freshly cut fruits and vegetables is intricate yet significant. Oxidative-damage-induced structural impairment and functional decline of cell membranes serve as the primary triggers for cellular senescence [48], which subsequently initiates a cascade of events that compromise quality. Specifically, increased membrane permeability disrupts cellular compartmentalization, increasing the likelihood of enzyme–substrate interactions and thereby exacerbating browning [49]. Furthermore, during the senescence of potato tuber tissue, increased amylase activity leads to starch degradation, resulting in reduced hardness [50]. Additionally, the depletion of ROS scavengers, such as ascorbic acid, contributes to nutrient loss in the product. While intact fruits and vegetables exhibit relatively slow accumulation of ROS during postharvest storage, fresh-cut processing induces a rapid increase in these species, accelerating oxidative stress-related events [51]. Plants mitigate oxidative damage via a sophisticated antioxidant system comprising various antioxidant compounds and enzymes. Key nonenzymatic antioxidants include ascorbic acid and polyphenols, whereas SOD, CAT, GR, and APX form the core enzymatic antioxidant network. These components collaborate through a series of chain reactions to scavenge superoxide anions and hydrogen peroxide, maintaining the cellular redox balance by eliminating free radicals [52]. The efficacy of this system is reflected in its ability to reduce the content of MDA, a marker of lipid peroxidation, and ion permeability, thereby safeguarding cell membrane integrity. The antioxidant properties of MT are well documented across various physiological processes, with substantial evidence supporting its role in fruits and fresh-cut tissues [13,53]. Research indicates that MT exerts its antioxidant effects through two mechanisms: direct scavenging of free radicals and activation of the antioxidant system [54,55]. Our research also revealed that MT maintains the content of nonenzymatic antioxidant substances in fresh-cut potatoes (Figure 5C,D) and enhances the activity of antioxidant enzymes (Figure 5E–H). Moreover, the increased free radical scavenging capacity and reduced malondialdehyde content and ion permeability (Figure 5I,J) further support the antioxidant effect of MT. Meanwhile, the inhibition of starch degradation (Figure 5A,B) implies a slowdown in the senescence process of fresh-cut potato cells under the action of MT. These results indicate that the effects of MT in delaying senescence exert a significant and indirect beneficial influence on the maintenance of color, firmness, and nutritional quality in fresh-cut potatoes. Importantly, the antioxidant effect is the result of synergistic interactions among various components, and individual antioxidant indicators may not exhibit strong correlations.

MT has been demonstrated to possess a specific receptor protein [56], a characteristic that supports its role as a primary messenger in modulating plant hormone signaling pathways and regulating the activity of certain transcription factors. In postharvest fruits such as apple [57], pear [58], and banana [59], MT has been shown to inhibit the synthesis of endogenous ethylene. Ethylene, a plant hormone, is known to accelerate the senescence of plant tissues and organs. Particularly in fresh-cut produce, the increase in ethylene levels postcutting stimulates the synthesis of enzymes such as amylase, pectinase, and Cx, which in turn accelerates tissue softening and increases the activity of PPO, thereby intensifying browning [60]. Therefore, exogenous MT application may increase the resistance of fresh-cut potatoes to traumatic stress and improve their quality by inhibiting ethylene synthesis. The transcription factor MYB168 is closely associated with lignin synthesis [61]. While no prior studies have specifically examined the response of MYB168 to MT, existing research on other members of the MYB gene family, such as MYB1, MYB2, and MYB108A [62,63], has demonstrated that MT exerts regulatory effects on some members of the MYB gene family. Therefore, the promotion of lignin synthesis in fresh-cut potatoes by MT may be attributable to its regulation of *StMYB168*. In addition, exogenous MT has been shown to promote endogenous MT synthesis in rice seedlings [64] and eggplants [65]. Our research indicates that this effect is also observed in fresh-cut potatoes. In this study, the optimal treatment dose (0.1 mmol/L) resulted in endogenous MT levels of 5–7 ng/g throughout the 12-day storage period (Figure 6D), remaining consistently below 10 ng/g, which is well within the typical range of MT content observed in natural foods [66]. This finding ensures the sustained upregulation of MT levels in fresh-cut potatoes during their shelf-life, which likely contributes to the prolonged efficacy of the treatment. To contextualize the efficacy of MT treatment, we compared our results with other natural preservatives reported for fresh-cut potatoes. Ascorbic acid (1%) reduced browning by 15–20% but had limited effects on firmness retention [67]. Citric acid (0.5%) combined with chitosan coating delayed browning by approximately 3 days but required complex application procedures. Tea polyphenol extract (0.5%) showed comparable antioxidant effects but exhibited pro-oxidant activity at higher concentrations. In comparison, MT at 0.1 mmol/L achieved simultaneous browning inhibition (8.13% reduction in BI) and firmness preservation (1.3-fold increase) without complex formulation requirements. Moreover, MT treatment activated endogenous biosynthetic pathways, creating a self-sustaining preservation effect absent in other treatments. These advantages position MT as a superior natural preservative for fresh-cut potatoes.

**Figure 8:**
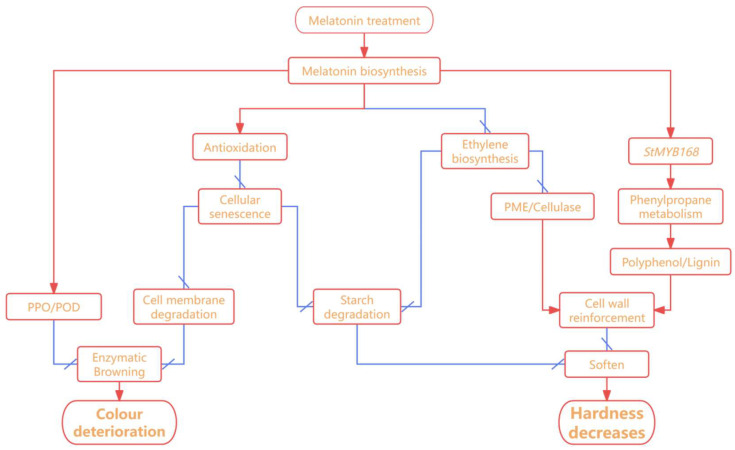
Multi-pathway mechanism of exogenous melatonin in maintaining fresh-cut potato quality. Red pointed arrows denote activation; blunt-end arrows denote inhibition.

In summary, the mechanism by which MT improves the quality of fresh-cut potatoes mainly involves multiple dimensions, with a focus on enhancing color and texture. The author has organized the potential correlations among the indicators involved in this article, as shown in Figure 8.

## 5. Conclusions

This study systematically elucidates the mechanism by which MT enhances the quality of fresh-cut potatoes from multiple perspectives, providing a robust theoretical foundation for the advancement of safer preservation technologies for fresh-cut fruits and vegetables. The study revealed that an optimal MT concentration of 0.1 mmol/L is suitable for fresh-cut potatoes. At this concentration, the color and firmness of fresh-cut potatoes are significantly improved, and their tissue structure becomes more stable. MT delays browning in fresh-cut potatoes by inhibiting the activities of PPO and POD while enhancing firmness by suppressing the degradation of cell wall polysaccharides and starch and activating phenylpropanoid metabolism to promote lignin synthesis. The improvement in the antioxidant capacity of fresh-cut potato cells induced by MT reduces the oxidative damage caused by free radicals, positively influencing both color and firmness. Under these conditions, the inhibition of ethylene biosynthesis and the activation of *StMYB168* represent key mechanisms through which MT mitigates browning and softening-related metabolism in fresh-cut potatoes. Exogenous MT treatment may sustain its beneficial effects by stimulating endogenous MT synthesis in fresh-cut potatoes. Based on the identified optimal melatonin concentration of 0.1 mmol/L and its regulatory mechanisms, melatonin-based strategies can be extended to other fresh-cut agricultural products to develop universal natural solutions for extending shelf life and improving safety.

## Figures and Tables

**Table 1:** Assay conditions for enzyme activity determination.

Enzyme.	Extraction Buffer ^1^	Reaction Mixture ^3^	U/ Temperature (°C)
PPO [19]	Acetate buffer (pH 5.5), 4% (*w*/*v*) PVP, 1% (*v*/*v*) Triton X-100	0.1 mL extract, 2.9 mL acetate buffer (pH 5.5, 50 mmol L^−1^ catechol)	1 × ΔA420·min^−1^/ 30 °C
POD [19]	Same as PPO	0.5 mL extract, 0.5 mL 0.5 mol L^−1^ H_2_O_2_, 2.0 mL acetate buffer (pH 5.5, 25 mmol L^−1^ guaiacol)	1 × ΔA470·min^−1^/ 37 °C
PME [19]	Citrate buffer (pH 5.5), 10% (*w*/*v*) NaCl	0.5 mL extract, 2.5 mL citrate buffer (pH 6.5, 0.1% (*w*/*v*) apple pectin, 50 mmol L^−1^ NaCl) ^4^	0.01 × ΔA420·h^−1^/ 37 °C
Cx [19]	Citrate buffer (pH 5.0) ^2^	0.5 mL extract, 1.5 mL 1% (*w*/*v*) CMC ^5^	1 μg reducing sugars per hour/ 37 °C
PAL [19]	Borax-hydroxide buffer (pH 9.8), 4% (*w*/*v*) PVP, 2 mmol L^−1^ EDTA, 5 mmol L^−1^ β-ME	0.5 mL extract, 3.0 mL borate buffer (pH 8.8), 0.5 mL 20 mmol L^−1^ L-phenylalanine	0.01 × ΔA290·min^−1^/ 40 °C
4CL [20]	Tris-HCl buffer (pH 8.0)	0.5 mL extract, 2.0 mL Tris-HCl buffer (pH 8.0, 5 mmol L^−1^ MgCl_2_, 1 mmol L^−1^ ATP, 12 µmol L^−1^ p-coumaric acid, 8 µmol L^−1^ CoA)	−0.01 × ΔA333·min^−1^/ 40 °C
C4H [21]	Tris-HCl buffer (pH 8.9)	0.5 mL extract, 2.5 mL Tris-HCl buffer (pH 8.0, 2 mmol L^−1^ trans-cinnamic acid, 5 µmol L^−1^ glucose-6-phosphate, 2 mmol L^−1^ NADP^+^)	−0.01 × ΔA340·min^−1^/ 30 °C
SOD [19]	Phosphate buffer (pH 7.5), 4% (*w*/*v*) PVP, 1% (*v*/*v*) Triton X-100, 5 mmol L^−1^ DTT	System of NBT method	50% NBT inhibition min^−1^/ 30 °C
CAT [19]	Same as SOD	0.2 mL extract, 2.8 mL phosphate buffer (pH 7.5, 10 mmol L^−1^ H_2_O_2_)	−1 × ΔA240·min^−1^/ 30 °C
APX [19]	SOD extraction buffer, 1 mmol L^−1^ AsA	0.1 mL enzyme extract, 2.6 mL Tris-HCl buffer (50 mmol L^−1^, pH 8.0, 0.1 mmol L^−1^ AsA), 0.3 mL 2.0 mmol L^−1^ H_2_O_2_	−1 × ΔA290·min^−1^/ 25 °C
GR [19]	Same as SOD	0.3 mL enzyme extract, 2.4 mL Tris-HCl buffer (pH 7.5, 0.5 mmol L^−1^ GSSG), 0.3 mL 3.5 mmol L^−1^ NADPH	−1 × ΔA340·min^−1^/ 25 °C

^1^: PVP, polyvinylpyrrolidone; β-ME, β-mercaptoethanol; DTT, dithiothreitol; AsA, ascorbic acid. ^2^: Tissue was sequentially extracted with 95% and 80% ethanol on ice, and the pellet was solubilized in extraction buffer (composition shown above). ^3^: CMC, carboxymethyl cellulose; NBT, nitroblue tetrazolium; and GSSG, oxidized glutathione. ^4^: Anthrone method for soluble sugars. ^5^: 3,5-dinitrosalicylic acid (DNS) method for reducing sugars.

## Data Availability

The original contributions presented in this study are included in the article. Further inquiries can be directed to the corresponding author.
